# The Challenges of Treating a *Helicobacter pylori* Infection following the COVID-19 Pandemic in Croatia: A Review

**DOI:** 10.3390/jcm13195762

**Published:** 2024-09-27

**Authors:** Ivana Jukic, Jonatan Vukovic

**Affiliations:** 1 Department of Gastroenterology and Hepatology, University Hospital Split, Spinciceva 1, 21000 Split, Croatia; jonatan.vukovic@mefst.hr; 2 Department of Health Studies, University of Split, 21000 Split, Croatia; 3 Department of Internal Medicine, School of Medicine, University of Split, 21000 Split, Croatia

**Keywords:** *H. pylori*, COVID-19, antibiotic resistance, antimicrobial stewardship, antibiotic consumption, eradication therapy

## Abstract

**Background:** *Helicobacter pylori* (*H. pylori*) is a prevalent bacterial pathogen implicated in different stomach and duodenal diseases, including chronic gastritis, gastric and duodenal ulcer, and stomach cancer. The COVID-19 pandemic has significantly influenced antibiotic prescription practices, potentially exacerbating antibiotic resistance in *H. pylori*. **Objective:** This study aims to investigate antibiotic prescription during the COVID-19 pandemic in Croatia and its possible impact on *H. pylori* antibiotic resistance, with a focus on Croatia. **Methods:** An extensive literature search was conducted to identify relevant studies from March 2020 to June 2024. The search strategy included terms related to COVID-19, antibiotic prescription, and Croatia. Studies were selected based on predefined eligibility criteria, focusing on observational research addressing antibiotic use during the pandemic. **Results:** The COVID-19 pandemic has led to significant fluctuations in community antibiotic consumption within the European Union/European Economic Area. In Croatia, antibiotic use in 2022 surpassed 2019 levels, driven by increased consumption of macrolides and other antibiotics. This surge, influenced by early pandemic treatment claims and penicillin shortages, contributed to rising antibiotic resistance in *H. pylori*. Resistance rates to clarithromycin and levofloxacin were notably high, driven by mutations in the 23S rRNA, gyrA, and gyrB genes. **Discussion:** The increased antibiotic use during the COVID-19 pandemic has highly likely complicated *H. pylori* eradication efforts, highlighting the necessity of judicious antibiotic use and robust antimicrobial stewardship. The pandemic underscored the need for new therapeutic strategies, optimized eradication regimens, and advanced diagnostic methods to manage bacterial infections effectively. **Conclusions:** The COVID-19 pandemic has significantly affected antibiotic use and resistance patterns, posing new challenges for *H. pylori* eradication. Addressing these challenges requires a multifaceted approach, including the development of new drugs and advanced diagnostics, coupled with sustained efforts in antimicrobial stewardship to combat emerging resistance threats.

## 1. Introduction

*Helicobacter pylori* (*H. pylori*) represents a ubiquitous bacterial pathogen, responsible for colonizing the gastric mucosa, and plays a pivotal role in the pathogenesis of various stomach and duodenal diseases, including chronic gastritis, gastric and duodenal ulcer, stomach adenocarcinoma, and mucosa-associated lymphoid tissue (MALT) lymphoma. The global prevalence of *H. pylori* infection is substantial, affecting over half of the world’s population, with marked variation between developed and developing regions due to differences in socioeconomic factors and hygiene standards [1,2].

Also, *H. pylori* infection has been identified as the most common infection-related cause of lethal outcome resulting from a malignant disease. The International Agency for Research on Cancer and the World Health Organization have designated *H. pylori* as a Group I carcinogen, recognizing it as the leading cause of gastric cancer, and early eradication of the bacteria, before the onset of metaplasia and atrophy, can effectively prevent the development of this cancer. Moreover, *H. pylori* eradication in high-risk regions decreases the risk of stomach cancer in asymptomatic individuals [3,4].

The eradication of *H. pylori* is complex, necessitating a combination of antibiotic therapy alongside proton pump inhibitors to enhance bacterial eradication and facilitate gastric mucosal healing. In general, the standard regimens depend on clarithromycin and metronidazole resistance rates. *H. pylori* eradication protocols typically comprise two or three antibiotics, selected from clarithromycin, amoxicillin, metronidazole, levofloxacin, or tetracycline, to circumvent resistance development [2].

However, the rising global prevalence of antibiotic-resistant *H. pylori* strains has significantly challenged eradication efforts, leading to the recommendation of tailored therapy based on local resistance patterns or the use of quadruple therapy as a first-line treatment in regions of high antibiotic resistance. The mechanism of resistance in *H. pylori* is multifactorial, involving chromosomal mutations that confer reduced susceptibility to antibiotics. Resistance rates exceed 15% for clarithromycin, range between 45% and 55between 45% to 55% for metronidazole, and between 14% and 20between 14% to 20% for levofloxacin [5]. Also, resistance to clarithromycin and levofloxacin primarily arises from point mutations in the bacterium’s genetic material, which alter the antibiotics’ target sites and interfere with drug activity [6].

In Croatia, an increase in primary resistance to these antibiotics in *H. pylori* has been observed, with mutations detected in the 23S rRNA, gyrA, and gyrB genes. These mutations alter target sites or protein structures, leading to resistance against clarithromycin and levofloxacin, and consequently reducing the effectiveness of treatment. Also, molecular docking analyses have shown that *H. pylori* strains harboring resistance-related mutations exhibit reduced susceptibility to clarithromycin and levofloxacin compared to wild-type strains, due to altered non-covalent interactions that weaken antibiotic–protein binding, leading to antibiotic resistance [7].

The COVID-19 pandemic is a global outbreak caused by the severe acute respiratory syndrome coronavirus 2 (SARS-CoV-2). The first cases of this novel coronavirus (nCoV) were detected in China in December 2019, with the virus rapidly spreading worldwide. In response, the World Health Organization (WHO) declared a Public Health Emergency of International Concern (PHEIC) on 30 January 2020 and subsequently characterized the outbreak as a pandemic on 11 March 2020. More than three years into the pandemic, on 5 May 2023, the WHO Emergency Committee on COVID-19 recommended to the Director-General that the disease, now well established and ongoing, no longer met the criteria for a PHEIC. This recommendation was accepted, signifying that the global emergency phase of the pandemic was over. However, it is important to note that this does not indicate the end of the pandemic itself, but rather the conclusion of its status as a global emergency [8].

The interrelation between COVID-19 management, particularly the use of antibiotics, and increased resistance in *H. pylori* has emerged as a notable concern. Research indicates that antibiotic regimens utilized in COVID-19 treatment may inadvertently foster antibiotic resistance in *H. pylori*, complicating subsequent eradication efforts [9].

This development underscores the necessity of judicious antibiotic use and highlights the broader implications of antimicrobial resistance in the context of a global pandemic. The scientific investigation into the relationship between COVID-19 infection and antibiotic resistance in *H. pylori* reveals intricate dynamics that are of significant concern to the medical community. Investigation has shown us that antibiotic treatments for *H. pylori* infections have shown lower-than-accepted eradication rates in patients previously treated for COVID-19, suggesting a link between the pandemic and increased antibiotic resistance in *H. pylori* infections [10].

The increased resistance rates to commonly used antibiotics in *H. pylori* eradication regimens call for a reevaluation of current treatment strategies and highlight the need for new medications, eradication protocols, and diagnostic tools such as next-generation sequencing to improve the control of *H. pylori* infections. The impact of COVID-19 on antibiotic resistance in *H. pylori* also emphasizes the importance of cautious antibiotic use during and beyond the pandemic to mitigate the risk of exacerbating resistance issues [10,11].

The goal of this review article was to determine the prescription of antibiotics during the COVID-19 pandemic in Croatia, as it may have an impact on the effectiveness of *H. pylori* eradication.

## 2. Materials and Methods

### 2.1. Information Source and Search Strategies

A scientific article search was accomplished in July 2024 to collect relevant studies addressing the review questions. For the period from the pandemic’s inception on 11 March 2020 to 30 June 2024, Medline electronic database (https://www.ncbi.nlm.nih.gov/pubmed) was searched comprehensively to identify relevant articles by using both free text terms and appropriate indexing terms. The basic search strategy employed was as follows: “COVID-19 AND ANTIBIOTIC AND PRESCRIPTION AND CROATIA”. The selection of studies was refined independently by two authors according to the predefined eligibility criteria. The articles retrieved from the electronic database were first screened for inclusion by reviewing the title and abstract. Subsequently, the full texts of studies that appeared potentially relevant were obtained and evaluated by the same two authors.

### 2.2. Eligibilty Criteria

The studies included in this review adhered to the following inclusion criteria: (1) original observational research, and (2) studies assessing antibiotic prescription practices during the COVID-19 pandemic for patients infected with SARS-CoV-2, as well as for other infectious diseases throughout the time of the COVID-19 pandemic period from March 2020 to May 2023. Specific inclusion criteria were as follows: studies involving human subjects only, encompassing an adult or young study population (without age restrictions), inclusive of both male and female participants, and addressing the diagnosis of acute COVID-19 infection or other infectious diseases during the pandemic. Exclusion criteria for this review were delineated as follows: (1) studies with primary endpoints not aligned with the scope of this review, (2) narrative reviews, commentaries, case reports, and case series, (3) in vitro or animal studies, (4) studies published in languages other than English, (5) articles without full-text availability, and (6) duplicate research publications.

## 3. Results

The scientific article search identified four (4) relevant titles and abstracts from the Medline database search. A total of four studies that met the full inclusion criteria for this review were retrieved and fully reviewed [12,13,14,15].

Bogdanic et al. [12] published a retrospective observational study and investigated azithromycin consumption in Croatia during 2020 and have made comparisons of their results to those for the time interval of 2017 to 2019. The authors examined data on azithromycin distribution covering the time interval from 1 January 2017 to 31 December 2020. Azithromycin consumption was quantified as days of therapy (DOT), calculated by dividing the total dose of azithromycin in a single package by the defined daily dose of 0.3 g. The data on azithromycin distribution to hospital and non-hospital pharmacies in Croatia were used as an approximate indicator of consumption. Authors have shown increased total azithromycin DOT distribution on an annual basis for the time interval from 2017 to 2020 (1.76, 1.91, 1.91, and 2.01/1000 inhabitant-days, respectively). From 2019 to 2020, this represented a 5.2% increase, and an 8.1% rise compared to the average distribution from 2017 to 2019. This 8.1% growth equates to a total of 37,224 azithromycin prescriptions, each for a 5-day course. Azithromycin was primarily distributed to non-hospital pharmacies, accounting for 93.2% to 95.6% of the total DOT. However, there was a notable upward trend in distribution to hospital pharmacies between 2017 and 2020, increasing from 4.4% to 6.8% of the total DOT over that period.

The total annual amount of azithromycin DOT units distributed was lowest in 2017 (2.670 million units) and highest in 2020 (2.976 million units), whereas the population-adjusted distribution was 642.85/1000 residents in 2017 and 733.43/1000 residents in 2020. The monthly distribution pattern of azithromycin in 2020 differed significantly from the previous three years. Azithromycin distribution to both hospital and non-hospital pharmacies in 2020 was highest in March, followed by November and December. The distribution of azithromycin to both hospital and non-hospital pharmacies in 2020 peaked in March, with subsequent high levels in November and December. Although the 8.1% increase in total azithromycin DOT from the 2017–2019 average to 2020 May seems modest, it is important to emphasize that Croatia is already among the countries with the highest percentage of macrolide resistant isolates in the EU/EEA [12,16].

Papst et al. [13] conducted a multicenter point-prevalence study in seven tertiary university hospitals (in medical divisions and intensive care units (ICUs)) in Croatia, Italy, Serbia, and Slovenia from 11 February 2021 to 15 April 2021. Of 988 COVID-19 patients, 521 were receiving antibiotics and/or antifungals (52.7%; range across hospitals: 32.9–85.6%) on the day of the study. Differences between hospitals were statistically significant (χ^2^ (6, *N* = 988) = 192.57, *p* < 0.001). Most of the patients were treated with antibiotics and/or antifungals within 48 hrs of admission (323/521, 62%; range across hospitals: 17.4–100%), their most common use was empirical (79.4% of prescriptions), and pneumonia was the main indication for starting the treatment (3/4 prescriptions). The majority of antibiotics prescribed (69.9%) fell under the “Watch” category of the World Health Organization (WHO) AWaRe classification, including 2nd–4th generation cephalosporins, antipseudomonal beta-lactams combined with beta-lactamase inhibitors, carbapenems, fluoroquinolones, macrolides, and vancomycin. In ICUs, antifungals and antibiotics from the “Reserve” group of the AWaRe classification (5th generation cephalosporins, polymyxins, glycylcyclins, oxazolidinones, lipopeptides, etc.) were prescribed more frequently than in medical divisions. Antifungals accounted for 11.6% of prescriptions, while “Reserve” group antibiotics made up 19.3% of ICU antimicrobial use. The pattern of antimicrobial prescriptions varied between hospitals. In some hospitals, antibiotics from the “Access” group (penicillins, beta-lactams with beta-lactamase inhibitors, tetracyclines, trimethoprim with sulfametoxazole, aminoglycosides, metronidazole, etc.) accounted for approximately one-third of prescriptions (including in ICUs), whereas in other hospitals their use was rare. The authors concluded that the overwhelming majority of prescriptions were empirical (79.4%), particularly in the early stages of hospitalization. Targeted treatment was more frequently observed in ICUs, likely due to the sampling of the lower respiratory tract [13].

Sokota et al. [14] reviewed the medical records of pediatric patients visiting emergency departments of four tertiary clinical hospital centers in Croatia, which were as follows: Dr Fran Mihaljević University Hospital for Infectious Diseases Zagreb (UHID), Children’s Hospital Zagreb (CHZ), University Hospital Center Split (UHC Split), and Clinical Hospital Center Rijeka (CHC Rijeka). Data were collected between 25 February and 25 April, 2018 and 2019, and between 25 February and 24 April 2020. Antimicrobial prescription was analyzed too. In 2020, the number of visits significantly declined across most diagnostic categories, with the most notable reduction observed in the category of infectious diseases.

The most frequently prescribed oral antibiotic was amoxicillin (n = 1160; 22.3%), followed by amoxicillin/clavulanate (n = 903, 17.3%), oral cephalosporins (n = 885, 17%), and azithromycin (n = 426, 8.2%). The structure of antibiotic prescribing remained unchanged during all the observed periods. The hospital with the greatest number of antibiotic prescriptions was UHID (n = 2073, 39.8%), followed by UHC Split (n = 1174, 22.5%), CHZ (n = 1042, 20%), and CHC Rijeka (n = 923, 17.7%). Azithromycin prescription was 3.4% in UHID, 7.1% in CHZ, 14.8% in UHC Split, and 11.6% in CHC Rijeka [14].

Sutej et al. [15] published retrospective observational research regarding the influence of the COVID-19 pandemic on prescribing trends in dental medicine in Croatia. Data on dental prescriptions and dispensed medications were sourced from the Croatian Health Insurance Fund (CHIF), the central government agency responsible for medical insurance. The basis for this analysis was all dental prescriptions funded by CHIF. Two year-intervals, from 2014/2015 to 2020/2021, were compared. Antimicrobial drugs (J01 and P01) were the most commonly prescribed medications, making up an average of 80% of all dental prescriptions. The first choice, as the most commonly prescribed antibiotic, was amoxicillin with clavulanic acid (co-amoxiclav), which accounted for an average of 64% of all prescriptions and 49% of all prescriptions. The total number of antibiotics prescribed by doctors of dental medicine increased by 7.744 (2.4%) in the first pandemic year (2020) and 6.222 (1.7%) in comparison to 2019. The most notable increase was observed for azithromycin, with a 39% rise in the first and 9% rise in the second pandemic year. However, this change was not statistically significant (*p* = 0.055). A statistically significant increase in utilization during the pandemic period was observed for co-amoxiclav (*p* = 0.002) for both pandemic years and clindamycin for the first pandemic year (*p* = 0.043). For cephalexin, a statistically significant decrease was observed during the pandemic period (*p* = 0.044) [15]. See Table 1.

## 4. Discussion

The COVID-19 pandemic has profoundly affected healthcare systems worldwide, leading to notable changes in antibiotic prescription practices. Notably, the observed patterns in community antibiotic consumption within the European Union/European Economic Area (EU/EEA) reflect these changes.

An international survey conducted in April 2020 among physicians treating patients with COVID-19 infection revealed that antibiotic prescribing practices were largely guided by clinical presentation, with a focus on covering atypical microorganisms. More than half of the respondents reported using a combination of β-lactams with either macrolides or fluoroquinolones [17].

The Council Recommendation adopted in June 2023 on stepping up EU actions against antimicrobial resistance in a One Health approach (2023/C 220/01) set an EU target of a 20% reduction in total antibiotic consumption (community and hospital sectors combined) by 2030, using 2019 as the baseline year (Council of the European Union. Council Recommendation on stepping up EU actions to combat antimicrobial resistance in a One Health approach) [18]. For the period between 2019 and 2022, Ventura-Gabarró C. et al. observed a high variability in the European Union/European Economic Area (EU/EEA) population-weighted mean community antibiotic consumption, in contrast to a continuously slow decrease observed between 2015 and 2019. Although there was an unprecedented 18.5% decrease in community consumption in 2020 compared with the 2019 baseline, this decrease appeared to be transient. Between 2021 and 2022, the EU/EEA mean community consumption increased by 18.8% and showed no significant difference from the pre-pandemic level in 2019. Moreover, in 13 of 27 countries, including Croatia, community antibiotic consumption was higher in 2022 than in 2019, with an average increase of 8.4% among these 13 countries. Increased consumption compared to baseline 2019 levels was recorded for Croatia in 2022 for antibacterials for systemic use, in 2022 for other beta-lactam antibacterials, in 2021 and 2022 for macrolides, lincosamides and streptogramins, and for other antibacterial groups in 2021 and 2022. At the EU/EEA level, the surge in consumption of ‘macrolides, lincosamides and streptogramins’ (ATC group J01F) in 2022 was largely driven by macrolide consumption and more particularly azithromycin (European Centre for Disease Prevention and Control (ECDC)) [19,20].

In response to media claims that azithromycin could be effective in treating COVID-19, its consumption surged in certain European countries, particularly during the early months of the pandemic [12,21,22].

Macrolides may have been used more frequently during the penicillin shortages reported in 2022, which could also account for the increased consumption of ‘other beta-lactam antibacterials’ (J01D, including cephalosporins) in some countries during that year [18,23].

The retrospective observational study by Bogdanic et al. [12] reveals significant insights into azithromycin consumption trends in Croatia during the COVID-19 pandemic. Notably, the study indicates an 8.1% increase in total azithromycin days of therapy (DOT) from the average of 2017–2019 to 2020. This rise, while modest, translates to a substantial 37,224 additional 5-day courses of azithromycin prescriptions. The pronounced increase in azithromycin distribution to hospital pharmacies (from 4.4% in 2017 to 6.8% in 2020) alongside a stable high distribution to non-hospital pharmacies suggests a shift possibly influenced by the pandemic’s healthcare demands. The monthly distribution pattern in 2020, peaking in March, November, and December, likely reflects the waves of COVID-19 infections and the corresponding surge in antibiotic use [12]. Papst et al.’s multicenter point-prevalence survey underscores the widespread use of antibiotics among COVID-19 patients across several countries, including Croatia. The significant variability in antibiotic prescription rates among hospitals (32.9% to 85.6%) points to differences in clinical practice and possibly varying degrees of adherence to antimicrobial stewardship principles. The predominance of empirical antibiotic use (79.4%) early in hospitalization highlights the urgent need for rapid diagnostic tools to guide targeted therapy. The frequent use of “Watch” group antibiotics aligns with global concerns about antimicrobial resistance, stressing the importance of cautious prescribing practices [13]. Sutej et al.’s study on dental prescriptions highlights an interesting facet of antibiotic use during the pandemic. Despite a general increase in antibiotic prescriptions by dentists, the significant rise in azithromycin use (39% in the first pandemic year) aligns with the trends observed in general healthcare settings. However, the lack of statistical significance (*p* = 0.055) suggests that this increase might not be entirely attributed to changes in clinical practice due to the pandemic. The significant increase in co-amoxiclav utilization underscores the need for continued surveillance and stewardship efforts to mitigate antimicrobial resistance risks [15]. Collectively, these studies paint a comprehensive picture of antimicrobial use in Croatia during the COVID-19 pandemic. The observed trends in azithromycin consumption, particularly the increase in hospital distribution and the peaks corresponding to COVID-19 waves, reflect the pandemic’s influence on prescribing practices. The high rates of empirical antibiotic use and the reliance on broad-spectrum agents underscore the challenges faced by healthcare providers in managing suspected bacterial infections amidst a viral pandemic. Given Croatia’s already high rates of macrolide-resistant isolates, as noted by the European Centre for Disease Prevention and Control, the increased azithromycin use is particularly concerning. It underscores the critical need for robust antimicrobial stewardship programs to balance the immediate demands of the pandemic with the long-term goal of preserving antibiotic efficacy.

So, in Croatia, the patterns mirrored broader EU/EEA trends, with the consumption of antibacterials for systemic use, including other beta-lactam antibacterials, macrolides, lincosamides, and streptogramins, as well as other antibacterial groups, significantly higher in 2022 compared to the 2019 baseline. This surge in antibiotic use, particularly macrolides like azithromycin, was influenced by early pandemic media claims suggesting azithromycin as a potential COVID-19 treatment. Additionally, reported penicillin shortages during the pandemic may have driven the increased consumption of other beta-lactam antibacterials, including cephalosporins. The increased use of antibiotics during the pandemic has important implications for antimicrobial resistance, particularly concerning *H. pylori*. The rise in antibiotic-resistant *H. pylori* strains poses a significant challenge to eradication efforts. For example, the standard regimen for *H. pylori* eradication, typically comprising two or three antibiotics alongside proton pump inhibitors, can be complicated by increasing resistance. According to the Maastricht VI/Florence consensus report, the first-line eradication therapy for *H. pylori* infection in Croatia is the bismuth quadruple therapy and if this therapy is not available then the first-line option is concomitant therapy [24]. Mestrovic et al. has concluded that 14-day concomitant and hybrid protocol treatments achieved very high but similar eradication rates in Croatia [25]. According to the randomized clinical trial conducted by Perkovic et al., tailored therapy based on antibiotic susceptibility testing shows a significantly higher eradication rate than the comparable empirical (concomitant) treatment. Therefore, tailored therapy could help achieve better eradication results and promote a personalized medicine approach to future patients [26].

Resistance rates exceed 15% for clarithromycin, range between 45% and 55% for metronidazole, and between 14% and 20% for levofloxacin, driven by chromosomal mutations that reduce antibiotic susceptibility [5]. In Croatia, primary resistance to these antibiotics in *H. pylori* has been rising, with mutations identified in the 23S rRNA, gyrA, and gyrB genes, affecting clarithromycin and levofloxacin resistance by modifying target sites or protein structures, diminishing treatment efficacy. Molecular docking analyses have shown that *H. pylori* strains harboring resistance-related mutations exhibit reduced susceptibility to clarithromycin and levofloxacin compared to wild-type strains, due to altered non-covalent interactions that weaken antibiotic–protein binding, leading to antibiotic resistance [7]. According to Tonkic et. al., overall resistance to azithromycin was 20.4%, and the same results were found for clarithromycin in Croatia [27].

The Council Recommendation adopted in June 2023 on strengthening EU efforts against antimicrobial resistance through a One Health approach (2023/C 220/01) established an EU target to reduce total antibiotic consumption (across both community and hospital sectors) by 20% by 2030, with 2019 as the baseline year [18]. The intersection of COVID-19 and *H. pylori* antibiotic resistance underscores the necessity of judicious antibiotic use. The pandemic has highlighted the broader implications of antimicrobial resistance, necessitating a reevaluation of current treatment strategies and the development of new drugs, eradication regimens, and diagnostic methods. The utilization of next-generation sequencing to identify resistance patterns and tailor antibiotic therapy is crucial in improving the control of *H. pylori* infections. Moreover, the COVID-19 pandemic’s influence on antibiotic resistance extends beyond *H. pylori*. The increased prescription of antibiotics during the pandemic, often as a precautionary measure against secondary bacterial infections, has likely contributed to the rise in resistant strains of various pathogens [28]. This phenomenon emphasizes the importance of antimicrobial stewardship programs and the development of comprehensive strategies to mitigate the risk of exacerbating resistance issues.

Prior to the onset of the COVID-19 pandemic, our research group focused on the challenge of *H. pylori* eradication in Croatia. We identified a critical gap in the knowledge base of general practitioners regarding the Maastricht Florence Consensus Report, which serves as the authoritative European guideline for the diagnosis and management of *H. pylori* infection. This knowledge deficit, particularly concerning the first and second empirical treatment regimens for *H. pylori*, significantly contributes to therapeutic failures and the subsequent exacerbation of antibiotic resistance [29].

Additionally, we documented insufficient access to key antibiotics that constitute the first-line therapeutic regimen for *H. pylori* in Croatia, further hindering eradication efforts [30]. The recommended first-line treatment in Croatia is the bismuth quadruple therapy, driven by the high prevalence of macrolide resistance. However, the COVID-19 pandemic witnessed a marked increase in macrolide prescriptions, which may have long-term implications for resistance patterns. The second-line empirical therapy typically involves fluoroquinolone-based triple or quadruple regimens. Given the heightened use of fluoroquinolones during the COVID-19 pandemic [13], we anticipate a future escalation in resistance rates, posing additional challenges to effective *H. pylori* eradication.

These findings underscore the growing complexity of *H. pylori* eradication, necessitating a shift in clinical responsibility from general practitioners to gastroenterologists. The increasing resistance trends will likely drive a greater reliance on personalized medicine approaches to optimize treatment outcomes in the face of evolving antibiotic resistance.

The limitations of this study are the heterogeneity of the population of each study (data from dental medicine, pediatric patients, hospitals, intensive care units, and the general population) and the small number of included studies. Also, the limitations are the lack of data for the comparison of results between countries, and that it is not a systematic review.

Our study provides new data regarding antibiotic consumption during the COVID-19 pandemic in Croatia and the challenges of the treatment of *H. pylori* infection.

## 5. Conclusions

The COVID-19 pandemic has significantly impacted antibiotic use and resistance patterns, posing new challenges for the eradication of *H. pylori.* Addressing these challenges requires a multifaceted approach that includes the development of new drugs, optimized eradication regimens, and advanced diagnostic methods. Ensuring the effective management of bacterial infections in the post-pandemic era will depend on sustained efforts in antimicrobial stewardship and innovative research to combat emerging resistance threats.

## 6. Future Directions

Regarding the increase in antibiotic prescriptions during the COVID-19 pandemic, it is necessary to investigate the primary, secondary, and tertiary resistance of *H. pylori* to antibiotics in Croatia and the world. Data on the antibiotic resistance of *H. pylori* are needed in order to be as effective as possible in the eradication of *H. pylori* and thus prevent further antibiotic resistance, which is a global and important public health problem.

## Figures and Tables

**Table 1:** Antibiotic prescription during COVID-19 pandemic in Croatia.

Authors/Year of Publication	Analyzed Period Study Population	Antibiotic/Group	Results
Bogdanic et al./2022 [12]	2017–2019, 2020, Croatian hospital and out-hospital population	azithromycin	A 5.2% increase from 2019 to 2020 and an 8.1% increase from the average of 2017–2019 to 2020
Papst et al./2022 [13]	2021, intensive care unit population	cephalosporins (2nd–4th generation), antipseudomonal beta-lactams with beta-lactamase inhibitors, carbapenems, fluoroquinolones, macrolides, and vancomycin.	Most prescriptions were empirical (79.4%)
Sokota et al./2021 [14]	2018, pediatric Croatian patients, 2019, 2020	amoxicillin amoxicillin/clavulanate oral cephalosporins azithromycin	The most frequently prescribed was amoxicillin (22.3%), followed by amoxicillin/clavulanate (17.3%), cephalosporins (17%), and azithromycin (8.2%).
Sutej et al./2023 [15]	2014/2015, dental patients, 2020/2021	amox. + clavulanic acid amoxicilin clindamycin metronidazole cephalexin azithromycin	The most prescribed was amoxicillin with clavulanic acid (64% of all prescriptions in 2020 and 49% of all prescriptions in 2021); the major increase was recorded for azithromycin in the first (39%) and second (9%) pandemic years
